# Zoledronic acid renders human M1 and M2 macrophages susceptible to Vδ2^+^ γδ T cell cytotoxicity in a perforin-dependent manner

**DOI:** 10.1007/s00262-017-2011-1

**Published:** 2017-05-13

**Authors:** Daniel W. Fowler, John Copier, Angus G. Dalgleish, Mark D. Bodman-Smith

**Affiliations:** Institute for Infection and Immunity, St. George’s University of London, Cranmer Terrace, Tooting, London, SW17 0RE UK

**Keywords:** γδ T cell, Vδ2^+^ T cell, Macrophage, Zoledronic acid, Cytotoxicity

## Abstract

Vδ2^+^ T cells are a subpopulation of γδ T cells in humans that are cytotoxic towards cells which accumulate isopentenyl pyrophosphate. The nitrogen-containing bisphosphonate, zoledronic acid (ZA), can induce tumour cell lines to accumulate isopentenyl pyrophosphate, thus rendering them more susceptible to Vδ2^+^ T cell cytotoxicity. However, little is known about whether ZA renders other, non-malignant cell types susceptible. In this study we focussed on macrophages (Mϕs), as these cells have been shown to take up ZA. We differentiated peripheral blood monocytes from healthy donors into Mϕs and then treated them with IFN-γ or IL-4 to generate M1 and M2 Mϕs, respectively. We characterised these Mϕs based on their phenotype and cytokine production and then tested whether ZA rendered them susceptible to Vδ2^+^ T cell cytotoxicity. Consistent with the literature, IFN-γ-treated Mϕs expressed higher levels of the M1 markers CD64 and IL-12p70, whereas IL-4-treated Mϕs expressed higher levels of the M2 markers CD206 and chemokine (C–C motif) ligand 18. When treated with ZA, both M1 and M2 Mϕs became susceptible to Vδ2^+^ T cell cytotoxicity. Vδ2^+^ T cells expressed perforin and degranulated in response to ZA-treated Mϕs as shown by mobilisation of CD107a and CD107b to the cell surface. Furthermore, cytotoxicity towards ZA-treated Mϕs was sensitive—at least in part—to the perforin inhibitor concanamycin A. These findings suggest that ZA can render M1 and M2 Mϕs susceptible to Vδ2^+^ T cell cytotoxicity in a perforin-dependent manner, which has important implications regarding the use of ZA in cancer immunotherapy.

**Electronic supplementary material:**

The online version of this article (doi:10.1007/s00262-017-2011-1) contains supplementary material, which is available to authorized users.

## Introduction

Human peripheral blood contains a subpopulation of γδ T cells that express TCRs composed of Vγ9 and Vδ2 subunits. These cells—referred to here as Vδ2^+^ T cells—typically represent 0.5–5% of peripheral blood T cells and exert potent cytotoxicity against their target cells.

Vδ2^+^ T cells detect intermediates of isoprenoid biosynthesis, namely isopentenyl pyrophosphate (IPP) and (E)-4-hydroxy-3-methyl-but-2-enyl pyrophosphate. IPP is generated by the endogenous mevalonate pathway as well as the exogenous 1-deoxy-d-xylulose-5-phosphate pathway, whereas (E)-4-hydroxy-3-methyl-but-2-enyl pyrophosphate is generated by the 1-deoxy-d-xylulose-5-phosphate pathway only [1]. The mevalonate pathway is often dysregulated in malignant and infected cells, resulting in accumulation of IPP and increased susceptibility to Vδ2^+^ T cell cytotoxicity [2, 3]. Moreover, certain cells accumulate IPP when exposed to the nitrogen-containing bisphosphonate (NBP), zoledronic acid (ZA) [4], a synthetic drug that inhibits an enzyme of the mevalonate pathway called farnesyl pyrophosphate synthase [5]. Although the precise mechanism of IPP and (E)-4-hydroxy-3-methyl-but-2-enyl pyrophosphate recognition by Vδ2^+^ T cells has yet to be determined, evidence suggests that it is TCR dependent and involves butyrophilin 3A1 [6].

Zoledronic acid is typically used to treat complications associated with excessive bone resorption in diseases such as osteoporosis, Paget’s disease and metastatic bone disease [7]. In terms of its mode of action, ZA binds to bone and disrupts the activity of bone remodelling cells called osteoclasts [8]. ZA also has potential as an immunotherapy for cancer, the proof of concept for which has already been demonstrated in clinical trials [9–11]. Although in cancer its mode of action is poorly understood, experiments in vitro have shown that tumour cell lines from a broad range of haematological and solid malignancies become more susceptible to Vδ2^+^ T cell cytotoxicity when exposed to ZA, suggesting a role for Vδ2^+^ T cells [12–14]. However, the capacity for ZA to induce susceptibility in other, non-malignant cell types is poorly characterised and could provide insight that helps to better understand the effects of this drug and improve its clinical application. In this study we have focussed on macrophages (denoted here as Mϕs) because these cells have been shown recently to take up NBPs in vivo [15] and are implicated in the progression of cancer [16].

Mϕs are tissue-resident phagocytic cells that play a critical role in tissue repair as well as immunity against pathogenic infection and malignant transformation [17]. Mϕs display functional plasticity that is intricately linked to their surrounding microenvironment [18]. Researchers have categorised the different functional states of Mϕs according to their capacity to either promote inflammation or suppress it. At one end of the spectrum are pro-inflammatory Mϕs, also referred to as M1 or classically activated Mϕs, and at the other end are anti-inflammatory Mϕs, also known as M2 or alternatively activated Mϕs [19]. IFN-γ and IL-4 have been identified as key drivers of these opposing M1 and M2 phenotypes, respectively [19].

As part of our ongoing studies into how ZA stimulates anti-tumour responses in Vδ2^+^ T cells, we identified a previously unexplored effect involving Vδ2^+^ T cell targeting of myeloid cells. Recently, we showed that ZA can render peripheral blood monocytes susceptible to Vδ2^+^ T cell cytotoxicity in vitro [20]. In a subsequent study by Junankar et al., tumour-associated Mϕs (TAMs) in breast cancer were identified as important targets for NBPs in vivo [15]. Therefore, we further explored the concept of Vδ2^+^ T cell targeting of myeloid cells, and found that ZA can render M1 and M2 Mϕs susceptible to Vδ2^+^ T cell cytotoxicity. Furthermore, we found that Vδ2^+^ T cell cytotoxicity towards ZA-treated Mϕs was dependent—at least in part—on perforin. This novel insight into the interplay between Vδ2^+^ T cells and Mϕs has important implications regarding the use of ZA in cancer immunotherapy.

## Materials and methods

### PBMC isolation

Anonymised leukocyte cones from healthy donors were obtained from the National Health Service blood transfusion unit at St. George’s Hospital, London. PBMCs were isolated by density-adjusted centrifugation using Histopaque-1077 (Sigma-Aldrich). RBCs were lysed with ammonium chloride solution and platelets removed by slow-speed centrifugation. PBMCs were resuspended at 2 × 10^7^ cells/ml of freezing medium (45% RPMI-1640, 45% FBS and 10% DMSO; all from Sigma-Aldrich) and frozen at −80 °C in Mr Frosty freezing containers (Thermo Scientific) prior to transferring them to liquid nitrogen.

### Cell culture

All cell culture was carried out in a humidified incubator at 37 °C with 5% CO_2_. To generate Mϕs, monocytes were isolated from PBMCs using CD14 microbeads according to the manufacturer’s instructions (Miltenyi Biotec). Monocytes were resuspended in serum-free medium (RPMI-1640 containing 2 mM l-glutamine, 100 units/ml penicillin and 100 μg/ml streptomycin; all from Sigma-Aldrich) at a density of 3.8 × 10^5^ cells/ml, and 200 μl, 2 or 5 ml of cell suspension added per well of 96-well, 12-well or 6-well tissue culture plates, respectively (Thermo Scientific). Monocytes were cultured for 2 h, after which time the majority of cells were adherent to the tissue culture plate. This process is known to activate monocytes and initiate the macrophage colony-stimulating factor production required for Mϕ differentiation [21]. The adherent monocytes were then cultured for 10 days in complete medium (RPMI-1640 containing 10% FBS, 2 mM l-glutamine, 100 units/ml penicillin and 100 μg/ml streptomycin), after which time the monocytes had differentiated into Mϕs, as indicated by the morphological changes and plastic adherence observed using light microscopy. M1 and M2 Mϕs were generated by adding 25 ng/ml of recombinant human IFN-γ or IL-4 (R and D Systems), respectively, on day 7. Mϕs that had not been treated with IFN-γ or IL-4 (designated M0s) were used as controls throughout. 10 μM ZA (Sigma-Aldrich) was added to the Mϕs on day 9. To generate pure populations of Vδ2^+^ T cells, PBMCs were resuspended at 2 × 10^6^ cells/ml of complete medium containing 1 μM ZA and 5 ng/ml recombinant human IL-2 (R and D Systems), and 250 μls of cell suspension added per well of 96-well round-bottomed tissue culture plates (Thermo Scientific). The cells were cultured for 9 days and fed every 2–3 days with fresh medium containing 5 ng/ml IL-2. Dead cells and non-γδ T cells were depleted sequentially using dead cell removal kits and TCRγδ negative isolation kits according to the manufacturer’s instructions (Miltenyi Biotec). The purity of Vδ2^+^CD3^+^ cells was assessed by flow cytometry using PE-conjugated mouse anti-human Vδ2 (clone 123R3; Miltenyi Biotec) and PerCP-conjugated mouse anti-human CD3 (clone SK7; Biolegend) or FITC-conjugated mouse anti-human CD3 (clone HIT3a; Becton–Dickinson). For one donor, a high percentage of Vδ1^+^CD3^+^ cells was detected post-isolation, and so Vδ1^+^ cells were depleted using allophycocyanin-conjugated recombinant human anti-Vδ1 (clone REA173; Miltenyi Biotec) and anti-allophycocyanin microbeads according to the manufacturer’s instructions (Miltenyi Biotec; supplementary Fig. 1). We speculate that this donor’s PBMCs had a particularly high percentage of Vδ1^+^ T cells prior to ZA and IL-2 stimulation and/or their Vδ1^+^ T cells underwent bystander expansion in response to ZA and IL-2.

### Flow cytometry

Day 10 Mϕs in six-well tissue culture plates were washed twice in PBS (Sigma-Aldrich) and cultured for 15 min in PBS containing 0.25% trypsin (Life Technologies) and 2 mM EDTA (Sigma-Aldrich). Cells were detached by repeated pipetting and then washed in complete medium to deactivate the trypsin. Mϕs were resuspended in flow cytometry buffer (PBS with 1% BSA and 0.09% sodium azide; all from Sigma-Aldrich) containing either FITC-conjugated mouse anti-human CD64 (clone 10.1; Becton–Dickinson) or PE-conjugated mouse anti-human CD206 (clone 19.2; Becton–Dickinson). Matched isotype controls were used to determine the amount of background expression. After 10 min at room temperature, cells were washed in flow cytometry buffer and fixed in CellFIX (Becton–Dickinson). Perforin expression in Vδ2^+^ T cells was assessed in PBMCs cultured with ZA and IL-2 for 0, 1 and 9 days as described in "Cell culture". Cells were resuspended in flow cytometry buffer containing PE-conjugated mouse anti-human Vδ2 (clone 123R3; Miltenyi Biotec) and PerCP-conjugated mouse anti-human CD3 (clone SK7; Biolegend). After 10 min at room temperature, cells were washed in flow cytometry buffer and simultaneously fixed and permeabilised using Cytofix/Cytoperm (Becton–Dickinson) according to the manufacturer’s instructions. Cells were washed and resuspended in Perm/Wash buffer (Becton–Dickinson) and then labelled with FITC-conjugated mouse anti-human perforin (clone δG9; Becton–Dickinson) or matched isotype controls. After 10 min at room temperature, cells were washed in Perm/Wash buffer and resuspended in flow cytometry buffer. Samples were acquired on an LSR II flow cytometer (Becton–Dickinson) and analysed using FlowJo software. All comparatively analysed samples were acquired on the same day except for the time course of perforin expression where day 0, 1 and 9 samples were acquired on different days. The mean fluorescence intensity (MFI) values stated throughout are arithmetic means.

### ELISAs

Day 10 Mϕs in 12-well tissue culture plates were washed twice in PBS and cultured overnight in complete medium (1 ml/well) with or without 100 ng/ml LPS (*E.coli* 0127:B8; Sigma-Aldrich). The concentration of IL-12p70 and chemokine (C–C motif) ligand (CCL) 18 within cell-free culture supernatants was determined using DuoSet ELISA kits according to the manufacturer’s instructions (R and D Systems). Optical densities at 450 nm were determined using a microplate reader (Dynex), and concentrations were extrapolated from standard curve data using a four parameter logistic model generated by GraphPad Prism 6 (GraphPad Software). Standard curves were 31.25–2000 pg/ml for IL-12p70, and 7.8125–500 pg/ml for CCL18.

### Carboxyfluorescein succinimidyl ester/Zombie-NIR cytotoxicity assay

Detaching the Mϕs from the tissue culture plates prior to performing the cytotoxicity assays resulted in poor viability; therefore, cytotoxicity was assessed by adding Vδ2^+^ T cells directly to adherent Mϕs. Day 10 Mϕs in 12-well tissue culture plates were washed twice in PBS and then cultured for 20 min in PBS containing 1 μM carboxyfluorescein succinimidyl ester (CFSE; Life Technologies). Mϕs were washed three times in complete medium and then cultured overnight with or without 1.52 × 10^6^ autologous Vδ2^+^ T cells per well in 2 ml complete medium to obtain an E:T ratio of 2:1 based on the initial seeding density of monocytes. For some experiments Vδ2^+^ T cells were pre-treated for 2 h with or without 100 ng/ml concanamycin A (CMA; Abcam) or DMSO, then washed three times in complete medium prior to being cultured with Mϕs. Non-adherent cells were collected and adherent cells detached from the tissue culture plates as described in “Flow cytometry”. All cells were washed in PBS and then labelled with Zombie-NIR live/dead cell discrimination dye according to the manufacturer’s instructions (Biolegend). Zombie-NIR binds to amine groups on proteins, but does not penetrate an intact plasma membrane. Live cells have relatively low expression because only cell surface proteins are available for binding, whereas dead cells exhibit higher levels of expression because their compromised plasma membrane permits binding to both extracellular and intracellular proteins. After 15 min at room temperature, cells were washed in complete medium and fixed in CellFIX. Samples were acquired on an LSR II flow cytometer and analysed using FlowJo software. All comparatively analysed samples were acquired on the same day.

### CD107 mobilisation assay

Day 10 Mϕs in 96-well tissue culture plates were washed three times in PBS and then cultured for 5 h with 1.52 × 10^5^ autologous Vδ2^+^ T cells per well in 200 μl complete medium to obtain an E:T ratio of 2:1 based on the initial seeding density of monocytes. Allophycocyanin-conjugated mouse anti-human CD107a (clone H4A3; Biolegend) and FITC-conjugated mouse anti-human CD107b (clone H4B4; Biolegend) or matched isotype controls were added directly to the wells at the start of the co-culture along with 1 μg/ml of monensin to neutralise intracellular acidity. Cells were then collected and labelled with PE-conjugated mouse anti-human Vδ2 (clone 123R3; Miltenyi Biotec) and PerCP-conjugated mouse anti-human CD3 (clone SK7; Biolegend) as described in “Flow cytometry”. Samples were acquired on an LSR II flow cytometer and analysed using FlowJo software. All comparatively analysed samples were acquired on the same day.

### Statistical analyses

**Fig. 1:**
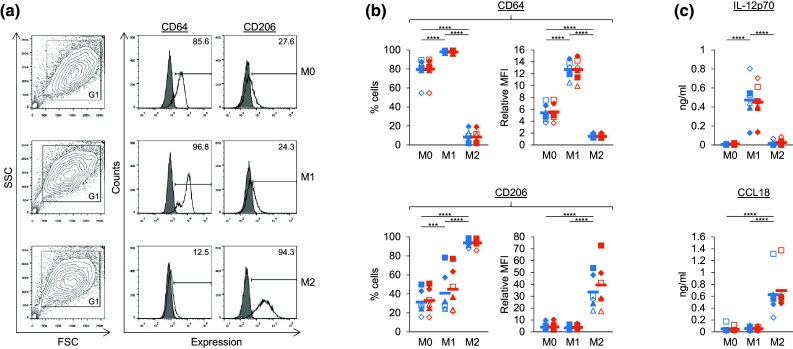
Characterisation of M1 and M2 Mϕs treated with or without ZA. **a**, **b** Flow cytometry was used to measure the expression of CD64 and CD206 on M0, M1 and M2 Mϕs treated with (*orange*) or without (*blue*) ZA for the last 18 h of culture. **a** Representative flow cytometry plots from one of six donors (−ZA). Dead cells and debris were excluded based on forward scatter (FSC) and side scatter (SSC) using gate (G) 1. Unfilled overlays = test, filled overlays = isotype. Numbers on the plots are percentage of cells within the marker. **b** Individual data points and means for six donors. Test MFIs for the total G1 population were divided by isotype controls to obtain relative MFIs. **c** M0, M1 and M2 Mϕs treated with (*orange*) or without (*blue*) ZA for the last 18 h of culture were cultured overnight in fresh medium with or without 100 ng/ml LPS. The concentration of IL-12p70 and CCL18 in culture supernatants was measured using ELISAs. Data for IL-12p70 is in the presence of LPS, whereas data for CCL18 is in the absence of LPS. Individual data points and means for six donors are shown. For **b** and **c**, data were analysed by repeated measures two-way ANOVA and comparisons between means carried out using Tukey’s multiple comparison tests. *** and **** indicate *p* values of <0.001 and <0.0001, respectively. Statistical differences for comparisons within the +ZA (*orange*) data sets are not shown

**Fig. 2:**
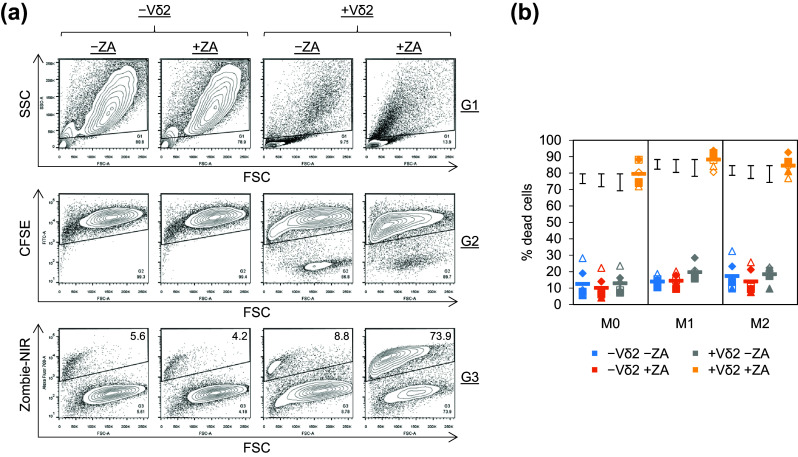
Vδ2^+^ T cell cytotoxicity towards ZA-treated M1 and M2 Mϕs. M0, M1 and M2 Mϕs treated with or without ZA for the last 18 h of culture were labelled with CFSE and cultured overnight with or without autologous Vδ2^+^ T cells. Flow cytometry was then used to measure Zombie-NIR expression. **a** Representative flow cytometry contour plots from one of six donors showing the gating strategy used to determine Zombie-NIR expression in M0 Mϕs. Mϕs were gated based on FSC and SSC using G1. CFSE^+^ cells within G1 were gated using G2. The percentage of Zombie-NIR^high^ cells (i.e. dead cells) within G1 + G2 was then determined using G3. Numbers on the contour plots are percentages of cells within G3. **b** Individual data points and means for six donors. Data were analysed by three-way ANOVA and comparisons between means carried out using Fisher’s LSD tests. The 5, 1 and 0.1% LSDs are depicted by the *black* intervals

**Fig. 3:**
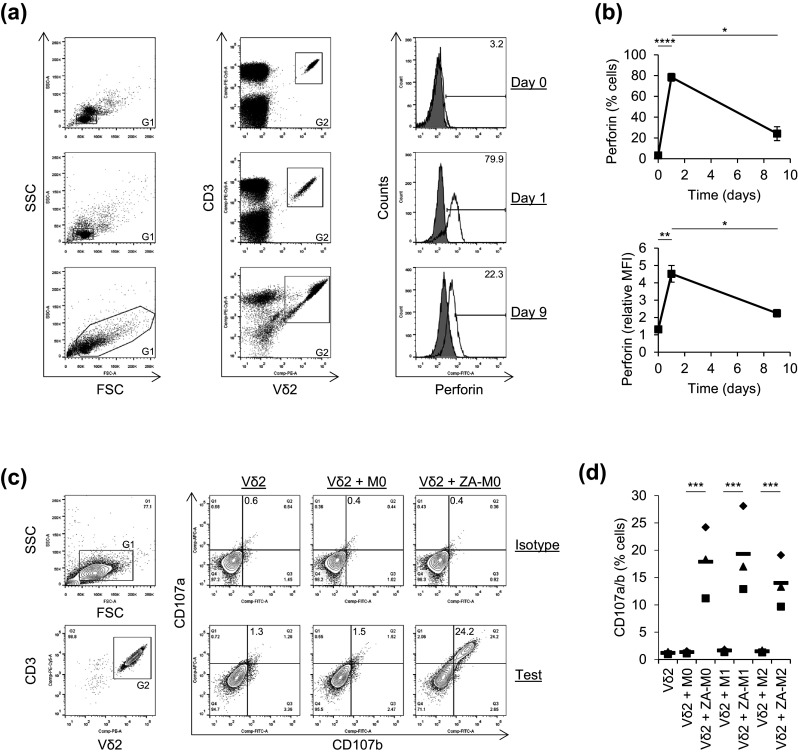
Expression of perforin and mobilisation of CD107a and CD107b in Vδ2^+^ T cells. **a**, **b** Flow cytometry was used to measure the expression of perforin by Vδ2^+^ T cells in PBMCs cultured with ZA and IL-2 for 0, 1 and 9 days. **a** Representative flow cytometry plots from one of three donors showing perforin expression in Vδ2^+^ T cells. Lymphocytes were gated based on FSC and SSC using G1. Note that G1 was extended at day 9 to incorporate blast cells. Vδ2^+^CD3^+^ cells within G1 were gated using G2. Percentage expression and MFI of perforin within G1 + G2 was then assessed. Unfilled overlays = test, filled overlays = isotype. Numbers on the histogram plots are percentage of cells within the marker. **b** Mean ± SD for three donors. Test MFIs for the total G1 + G2 population were divided by the isotype controls to obtain relative MFIs. Data were analysed by repeated measures one-way ANOVA and comparisons between means carried out using Tukey’s multiple comparison tests. **c**, **d** Vδ2^+^ T cells were cultured with or without autologous M0, M1 or M2 Mϕs that had been treated with or without ZA for the last 18 h of culture. Flow cytometry was used to measure the expression of CD107a and CD107b by Vδ2^+^ T cells. **c** Representative flow cytometry contour plots from one of three donors showing CD107a and CD107b expression on gated Vδ2^+^CD3^+^ cells. Lymphocytes were gated based on FSC and SSC using G1. Vδ2^+^CD3^+^ cells within G1 were gated using G2. Percentage expression of CD107a and CD107b within G1 + G2 was then assessed. Quadrants were set against the Vδ2 alone controls, and separate quadrants were generated for isotype and test. Numbers are percentages of cells contained within the *upper right* quadrants. **d** Individual data points and means for three donors. Data were analysed by repeated measures two-way ANOVA and comparisons between means carried out using Sidak’s multiple comparison tests. For **b** and **d**, *, **, *** and **** indicate *p* values of <0.05, <0.01, <0.001 and <0.0001, respectively

**Fig. 4:**
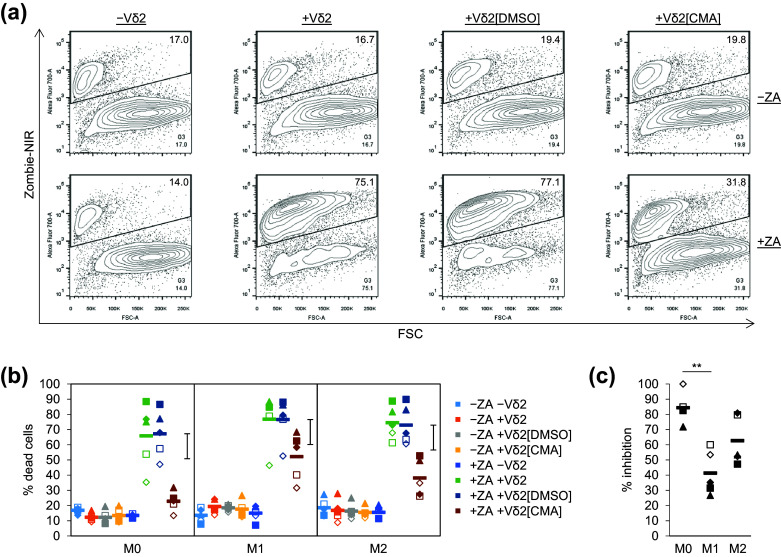
The effect of concanamycin A on Vδ2^+^ T cell cytotoxicity towards ZA-treated Mϕs. M0, M1 and M2 Mϕs treated with or without ZA for the last 18 h of culture were labelled with CFSE and then cultured overnight with or without autologous Vδ2^+^ T cells that had been pre-treated for 2 h with or without CMA (100 ng/ml) or DMSO. Flow cytometry was then used to measure Zombie-NIR expression. **a** Representative flow cytometry contour plots for M0 Mϕs from one of five donors. The percentage of Zombie-NIR^high^ cells (i.e. dead cells) within CFSE^+^ Mϕs was determined using the G1 + G2 + G3 gating strategy described in Fig. 2. Numbers on the plots are percentages of cells within G3. **b** Individual data points and means for five donors. Data were analysed by three-way ANOVA and comparisons between means carried out using Fisher’s LSD tests. The 0.1% LSD is depicted by the *black* interval. **c** Data in **b** was expressed as percentage inhibition. Within the +ZA data sets, the percentage of dead Mϕs in the absence of Vδ2^+^ T cells (i.e. background cell death) was subtracted from that induced by the DMSO- and CMA-treated Vδ2^+^ T cells. The corrected values for Mϕ cell death induced by CMA-treated Vδ2^+^ T cells were then expressed as a percentage of the corrected values for Mϕ cell death induced by DMSO-treated Vδ2^+^ T cells. These values were then converted to percentage inhibition by subtracting them from 100%. Data were analysed by repeated measures one-way ANOVA and comparisons between means carried out using Tukey’s multiple comparison tests. ** Indicates a *p* value <0.01

Data in Figs. 1b, c, 3b, d and 4c were analysed by repeated measures one-way or two-way ANOVA and comparisons between means carried out using either Tukey’s or Sidak’s multiple comparison tests (GraphPad Prism 6). *, **, *** and **** were used to indicate *p* values of <0.05, <0.01, <0.001 and <0.0001, respectively. Gaussian distributions were assumed. Data in Fig. 2b included a three-way (3 × 2 × 2) factorial design repeated six times using cells from six different donors. The three factors were Mϕ type (M0, M1 and M2), ±ZA and ±Vδ2 cells. Data in Fig. 4b were a three-way (3 × 2 × 4) factorial design repeated five times using cells from five different donors. The three factors were Mϕ type (M0, M1 and M2), ±ZA and ±Vδ2 cells (−Vδ2, +Vδ2, +Vδ2[DMSO] and +Vδ2[CMA]). Data in Figs. 2b and 4b were analysed by three-way ANOVA and comparisons between means carried out using Fisher’s least significant difference (LSD; Genstat 18). Assumptions underlying the analysis were checked using the diagnostic plots produced by the software. LSDs at the 5, 1 and 0.1% level are depicted by black intervals, and differences in the means that were greater than this interval were deemed significant to an equivalent *p* value of <0.05, <0.01 and <0.001, respectively.

## Results

### ZA did not alter M1 or M2 markers on Mϕs

We differentiated monocytes from the peripheral blood of healthy donors into Mϕs and treated them with IFN-γ or IL-4 to generate M1 and M2 Mϕs, respectively. We then characterised these Mϕs based on their expression of markers for M1 Mϕs (CD64 and IL-12p70) and M2 Mϕs (CD206 and CCL18) [19, 22]. M1 Mϕs had upregulated expression of CD64, whereas M2 Mϕs had downregulated CD64 and upregulated CD206 (Fig. 1a, b). Although, statistically, we observed significantly higher levels of CD206 on M1 Mϕs compared with M0 Mϕs in terms of percentage expression, this was not consistent for all donors and not statistically significant in terms of relative MFI (Fig. 1b). Mϕs were then cultured overnight with or without LPS to measure the production of IL-12p70 and CCL18, respectively. M1 Mϕs produced more IL-12p70 than M0 and M2 Mϕs, whereas M2 Mϕs produced more CCL18 than M0 and M1 Mϕs (Fig. 1c). We also tested whether ZA—added for the last 18 h of culture—had any effect on these markers and found little or no difference between untreated and ZA-treated Mϕs (Fig. 1). Taken together, these data validate our protocol for generating M1 and M2 Mϕs and show that ZA does not alter the M1 and M2 profile of the Mϕs in this system.

### ZA-rendered M1 and M2 Mϕs susceptible to Vδ2^+^ T cell cytotoxicity

To obtain sufficient cell numbers for cytotoxicity assays, we stimulated Vδ2^+^ T cell expansion prior to isolation. Vδ2^+^ T cell expansion was observed in PBMCs treated with ZA and IL-2 for 9 days, as shown by increased frequencies of Vδ2^+^CD3^+^ cells (supplementary Fig. 2). Vδ2^+^ T cells were purified by sequentially depleting dead cells and non-γδ T cells (mean ± standard deviation (SD) for the percentage of Vδ2^+^CD3^+^ cells from four donors = 97.2 ± 1.8; supplementary Fig. 2). The percentage of Vδ2^+^CD3^+^ cells at day 0 and day 9 pre-depletion of dead cells and non-γδ T cells was not assessed routinely; however, purities at day 9 post-depletion were assessed for all isolations performed in this study (mean ± SD for 14 isolations = 97.7 ± 1.8). We conducted preliminary experiments to determine the optimal E:T ratio and ZA concentration for Vδ2^+^ T cell-mediated cytotoxicity against ZA-treated Mϕs (supplementary Fig. 3). These experiments showed Vδ2^+^ T cell cytotoxicity and degranulation against Mϕs treated with 10 μM, but not 1 μM ZA (supplementary Fig. 3). Furthermore, they showed marked killing at the lowest E:T ratio of 2:1 (supplementary Fig. 3). Using the 10 μM concentration of ZA and 2:1 E:T ratio, we found that ZA had little or no effect on Mϕ viability in the absence of Vδ2^+^ T cells, and Vδ2^+^ T cells did not induce cell death in Mϕs that had not been treated with ZA (Fig. 2a, b). However, there was a marked increase in the amount of cell death in Mϕs that were pre-treated with ZA and then cultured with Vδ2^+^ T cells (Fig. 2a, b). Although, statistically, Vδ2^+^ T cell-mediated killing of ZA-treated M1 Mϕs was significantly higher than that of M0 Mϕs, the difference was relatively small and no statistically significant difference was found between M1 and M2 Mϕs (Fig. 2b). These results suggest that Vδ2^+^ T cells are cytotoxic towards ZA-treated Mϕs irrespective of their M0, M1 and M2 phenotype.

### Vδ2^+^ T cells expressed perforin and degranulated when cultured with ZA-treated Mϕs

Perforin has been shown previously to play a role in γδ T cell cytotoxicity towards tumour cell lines [23, 24]; therefore, we tested whether perforin contributes to Vδ2^+^ T cell cytotoxicity towards ZA-treated Mϕs. We measured perforin expression by Vδ2^+^ T cells before, during and after expansion with ZA and IL-2. We found that, although resting Vδ2^+^ T cells expressed little or no perforin, it was markedly upregulated after 1 day of culture with ZA and IL-2 (Fig. 3a, b). After 9 days of culture with ZA and IL-2, perforin was downregulated but still expressed by Vδ2^+^ T cells (Fig. 3a, b). We also measured perforin expression in expanded and isolated Vδ2^+^ T cells from six donors and found consistent expression in terms of percentage expression (mean ± SD = 1.2 ± 0.5 vs. 23.9 ± 7.6 for isotype and test, respectively) and MFI (mean ± SD = 227.8 ± 15.2 vs. 490.7 ± 104.6 for isotype and test, respectively). To determine if Vδ2^+^ T cells release perforin when cultured with ZA-treated Mϕs, we measured the mobilisation of lysosomal-associated membrane protein 1 and 2 (i.e. CD107a and CD107b) to the surface of Vδ2^+^ T cells. CD107a—and to a lesser extent CD107b—was expressed on Vδ2^+^ T cells that were isolated from PBMCs after 9 days of culture with ZA and IL-2 (Fig. 3c). This may represent residual CD107 expression from the monocyte-dependent degranulation that is induced when PBMCs are exposed to ZA [20]. Vδ2^+^ T cells upregulated expression of CD107a and CD107b on their cell surface when cultured with ZA-treated Mϕs compared with untreated Mϕs (Fig. 3c, d). These data suggest that Vδ2^+^ T cells express perforin and degranulate in response to ZA-treated Mϕs, thus implicating a role for perforin in Vδ2^+^ T cell cytotoxicity towards ZA-treated Mϕs.

### Vδ2^+^ T cell cytotoxicity towards ZA-treated Mϕs was sensitive to concanamycin A

To explore further the potential role of perforin in Vδ2^+^ T cell cytotoxicity towards ZA-treated Mϕs, we repeated the cytotoxicity assays shown in Fig. 2, but this time pre-treated Vδ2^+^ T cells with the H^+^-ATPase inhibitor CMA. CMA blocks acidification of cytolytic granules, which inhibits perforin-mediated but not Fas ligand-mediated cytotoxicity [25]. We found that pre-treating Vδ2^+^ T cells with CMA reduced their cytotoxicity towards Mϕs compared with DMSO controls (Fig. 4a, b). We calculated the percentage inhibition for Vδ2^+^ T cell cytotoxicity towards M0, M1 and M2 Mϕs and found that Vδ2^+^ T cell cytotoxicity towards M0 Mϕs was more sensitive to CMA than towards M1 Mϕs (Fig. 4c). To determine whether CMA had an effect on Vδ2^+^ T cell viability, we applied a gate to CFSE^−^ cells and calculated the percentage of Zombie-NIR^low^ cells (supplementary Fig. 4a). There was a discernible reduction in Vδ2^+^ T cell viability in the presence of ZA-treated Mϕs compared with untreated Mϕs; however, there was little or no difference in Vδ2^+^ T cell viability between the CMA and DMSO treatment groups (supplementary Fig. 4b). These findings suggest that Vδ2^+^ T cell cytotoxicity towards ZA-treated Mϕs is sensitive—at least in part—to CMA, thus implicating a role for perforin.

## Discussion

Vδ2^+^ T cells in the peripheral blood of humans are regarded as sentinels against infection [26] and malignant transformation [27]. They express the inflammatory homing receptors chemokine (C–C motif) receptor 5 and chemokine (C–X–C motif) receptor 3 [28] and thus infiltrate sites of infection [29] as well as the inflammatory microenvironment of diseased tissues such as tumours [30, 31]. Mϕs are abundant in these tissues and are likely to interact closely with infiltrating Vδ2^+^ T cells. We explored the potential interaction between Vδ2^+^ T cells and Mϕs in vitro and found that ZA can render M1 and M2 Mϕs susceptible to Vδ2^+^ T cell cytotoxicity in a perforin-dependent manner.

Zoledronic acid has a high affinity for hydroxyapatite [32] and thus binds rapidly to bone following i.v. infusion [33]. Therefore, the Mϕs most likely to be exposed to ZA are those associated with bone and/or the surrounding tissues: for example, the TAMs in bone-related cancers such as osteosarcoma, myeloma and secondary bone metastases associated with cancers of the prostate, lung and breast. Following i.v. infusion, NBPs may also reach tissues other than bone. Intravital imaging in a murine model of breast cancer showed that a fluorescently labelled NBP—given by i.v. injection—leaked from the vasculature of mammary tumours and bound rapidly to granular microcalcifications, which were subsequently engulfed by TAMs [15]. The NBP was not retained in cells other than Mϕs, nor was it retained in B16 tumours, which lack microcalcifications [15]. This study suggests that calcified tissues other than bone can also accumulate NBPs [15]. The lack of cytotoxicity and degranulation at 1 μM ZA that was observed in our preliminary optimisation experiments suggests that the Mϕs most likely to be targeted by Vδ2^+^ T cells following ZA treatment are those associated with calcified tissues where the drug is likely to accumulate, which has important implications regarding the in vivo effects of this drug. It is worth noting that uptake of ZA by Mϕs in vivo may be markedly different using other methods of delivery such as liposome or nanoparticle encapsulation [34, 35] and localised injection. At the cellular level, experiments conducted in vitro suggest that ZA is taken up by myeloid cells such as monocytes, Mϕs and osteoclasts via the process of fluid phase endocytosis [36, 37].

Zoledronic acid inhibits farnesyl pyrophosphate synthase of the mevalonate pathway, which has been shown in vitro to induce apoptosis directly in the murine Mϕ-like cell line J774.2 [38]. A potential mechanism for this effect is accumulation of the pro-apoptotic analogue of ATP, Apppl, which has been reported to accumulate in ZA-treated cells such as osteoclasts and MCF-7 cells [4]. Interestingly, ZA did not affect the viability of Mϕs in our experiments; however, we used relatively short exposure times and did not look at markers of early stage apoptosis such as surface expression of phosphatidyl serine. Inhibition of farnesyl pyrophosphate synthase may also modulate the differentiation and function of Mϕs. For example, when monocyte-derived M2 Mϕs were differentiated in the presence of ZA, they had reduced expression of CD206 and IL-10, and an impaired capacity to promote angiogenesis and tumour cell invasion [39]. ZA also inhibited tumour growth in a murine model of cervical cancer, which correlated with reduced angiogenesis and decreased production of matrix metallopeptidase 9 by Mϕs proximal to and associated with tumours [40]. Furthermore, ZA reduced the onset and growth of tumours in a murine model of breast cancer, which correlated with reduced vascularisation of the tumour, reduced numbers of TAMs, and repolarisation of TAMs from an M2 to M1 phenotype [41]. Taken together, these studies suggest that ZA can modulate the differentiation of Mϕs towards an M1 phenotype. To the best of our knowledge, Vδ2^+^ T cell targeting of ZA-treated M1 and M2 Mϕs—as suggested by our data—has been previously unreported and broadens our understanding of the effects of ZA on Mϕs. Importantly, mice do not develop the Vδ2^+^ T cell subset that responds to ZA-induced accumulation of IPP because they lack the gene for butyrophilin 3A1 [42], thus highlighting the importance of using human cells for this study.

Our data suggests that ZA has the potential to kill M1 and M2 Mϕs indirectly within tissues that are exposed to the drug and infiltrated by Vδ2^+^ T cells. Tumours contain an abundant population of Mϕs, which typically express M2 markers and correlate with a poor prognosis [43]. In breast cancer, CCL18 production by TAMs promotes angiogenesis and thus supports tumour growth and dissemination [44]. Furthermore, M2 Mϕs in the bone marrow of multiple myeloma patients have been shown to protect malignant cells from chemotherapy-induced apoptosis [45, 46]. In contrast, osteosarcomas can contain relatively high percentages of M1 Mϕs, which are associated with reduced metastases and improved survival [47]. The potential for ZA to render Mϕs susceptible to Vδ2^+^ T cells may be beneficial or detrimental depending on which type of Mϕ is present in the tumour. For example, it may be beneficial in patients with breast cancer or myeloma and could explain the promising responses to ZA reported for clinical trials in these cancer types [48, 49], whereas it may be counterproductive in osteosarcoma.

It is important to note that our study has focussed on the killing capacity of activated Vδ2^+^ T cells. Although it would be interesting to compare the cytotoxicity of resting and activated Vδ2^+^ T cells, the relatively low frequency of Vδ2^+^ T cells in peripheral blood meant that we were unable to isolate the number of resting Vδ2^+^ T cells required to perform the cytotoxicity assays used in this study. Whether or not i.v. infusion of ZA—combined with i.v. or s.c. IL-2—can activate peripheral blood Vδ2^+^ T cells in vivo is a point of contention. Current hypotheses state that peripheral blood monocytes take up ZA following i.v. infusion and subsequently activate Vδ2^+^ T cells [37]; indeed, proliferation and/or differentiation of peripheral blood Vδ2^+^ T cells has been reported in some patients receiving ZA and IL-2 [9–11]. However, Vδ2^+^ T cell responses were not observed in all patients [50] and it is unclear whether this is due to lack of activation or detection. Importantly, Vδ2^+^ T cells that are pre-activated may be more cytotoxic than resting, and thus ZA-induced targeting of Mϕs by Vδ2^+^ T cells in vivo may be suboptimal in patients for whom ZA and IL-2 treatment fails to activate their circulating Vδ2^+^ T cells, thus highlighting the importance of effective Vδ2^+^ T cell priming in the periphery.

In our study, Vδ2^+^ T cell cytotoxicity towards M0, M1 and M2 Mϕs was sensitive—at least in part—to the perforin inhibitor CMA, thus implicating a role for perforin [25]. Interestingly, CMA did not inhibit cytotoxicity completely, and the degree of inhibition varied between the different types of Mϕ; specifically, Vδ2^+^ T cell cytotoxicity towards M0 Mϕs was more sensitive to CMA than towards M1 Mϕs. If, in our assays, CMA blocked perforin completely, our data would suggest that other mechanisms of cell-mediated cytotoxicity are involved and that the contribution of perforin versus other mechanisms of cytotoxicity varies between the different types of Mϕ. Indeed, Vδ2^+^ T cells have been shown to kill target cells through the expression of Fas ligand and TRAIL [51]. However, if perforin blockade was incomplete, the variation in sensitivity to CMA that was observed between the different types of Mϕ could also be attributed to differences in their susceptibility to perforin-mediated killing under conditions of suboptimal perforin activity. Nonetheless, our data suggest that perforin plays a role, which provides a useful mechanistic marker for exploring this concept in vivo.

In conclusion, this study sheds light on a potential interaction between Vδ2^+^ T cells and Mϕs following ZA treatment and suggests a mechanism of action for this drug that may help its future development in cancer immunotherapy.

### Electronic supplementary material

Below is the link to the electronic supplementary material.

Supplementary material 1 (PDF 538 kb)

## Abbreviations

CCL: Chemokine (C–C motif) ligand
CFSE: Carboxyfluorescein succinimidyl ester
CMA: Concanamycin A
FSC: Forward scatter
G: Gate
IPP: Isopentenyl pyrophosphate
LSD: Least significant difference
Mϕ: Macrophage
MFI: Mean (arithmetic) fluorescence intensity
NBP: Nitrogen-containing bisphosphonate
SD: Standard deviation
SSC: Side scatter
TAM: Tumour-associated macrophage
ZA: Zoledronic acid
