# Supplementary material for: Zoledronic acid renders human M1 and M2 macrophages susceptible to Vδ2+ γδ T cell cytotoxicity in a perforin-dependent manner
Source: Cancer Immunol Immunother. 2017 May 13;66(9):1205–15. doi: 10.1007/s00262-017-2011-1 (PMC5579165; doi:10.1007/s00262-017-2011-1)
Supplement: Supplementary file 1 — Supplementary material 1 (PDF 538 kb) [file 262_2017_2011_MOESM1_ESM.pdf]

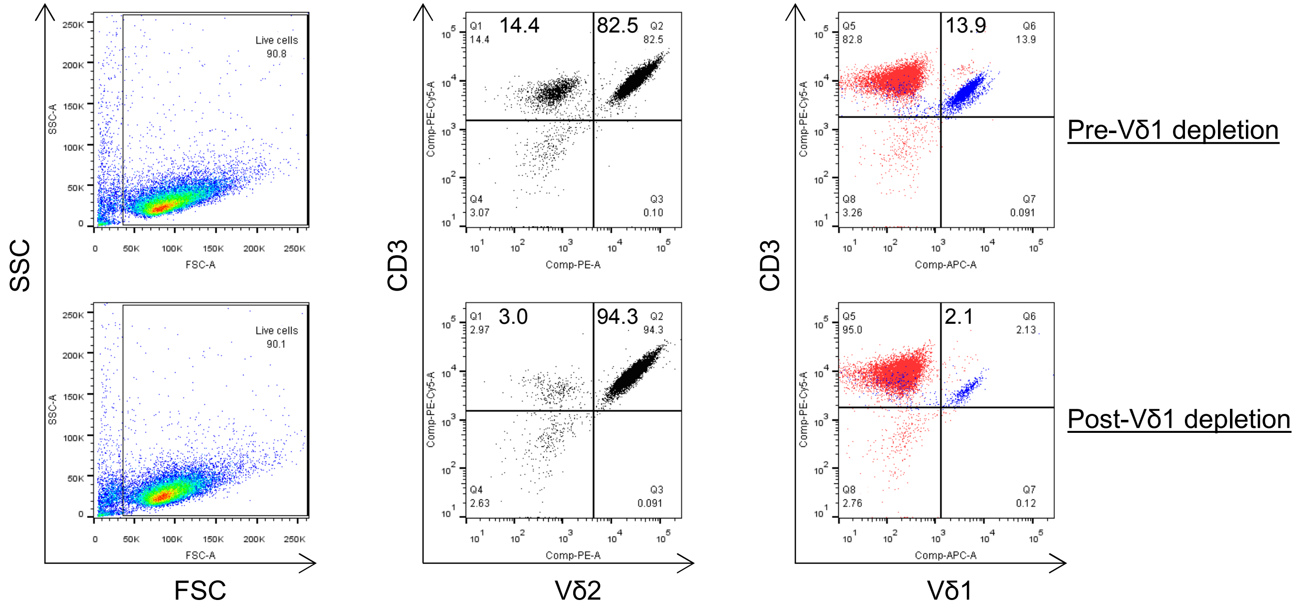

Supplementary Fig. 1: The additional Vδ1$^+$ T cell depletion step used to increase the purity of Vδ2$^+$ T cells from one of the donors. PBMCs were treated for 9 days with 1μM ZA and 5ng/ml IL-2. Culture medium was replaced with fresh medium containing 5ng/ml IL-2 every 2–3 days. Dead cells, non-γδ T cells and Vδ1$^+$ cells were depleted sequentially using MACS. Flow cytometry was used to measure the percentage of Vδ2$^+$CD3$^+$ and Vδ1$^+$CD3$^+$ cells pre- and post-Vδ1$^+$ cell depletion. Flow cytometry plots are from one donor. Dead cells and debris were excluded based on FSC and SSC, and Vδ2, Vδ1 and CD3 expression was assessed on gated cells. Numbers on the dot plots are percentages of cells contained within the quadrants. Vδ2$^-$CD3$^+$ cells (i.e. cells in the upper left quadrant of the middle column of dot plots) were coloured blue on the Vδ1 vs. CD3 dot plots on the right hand side.

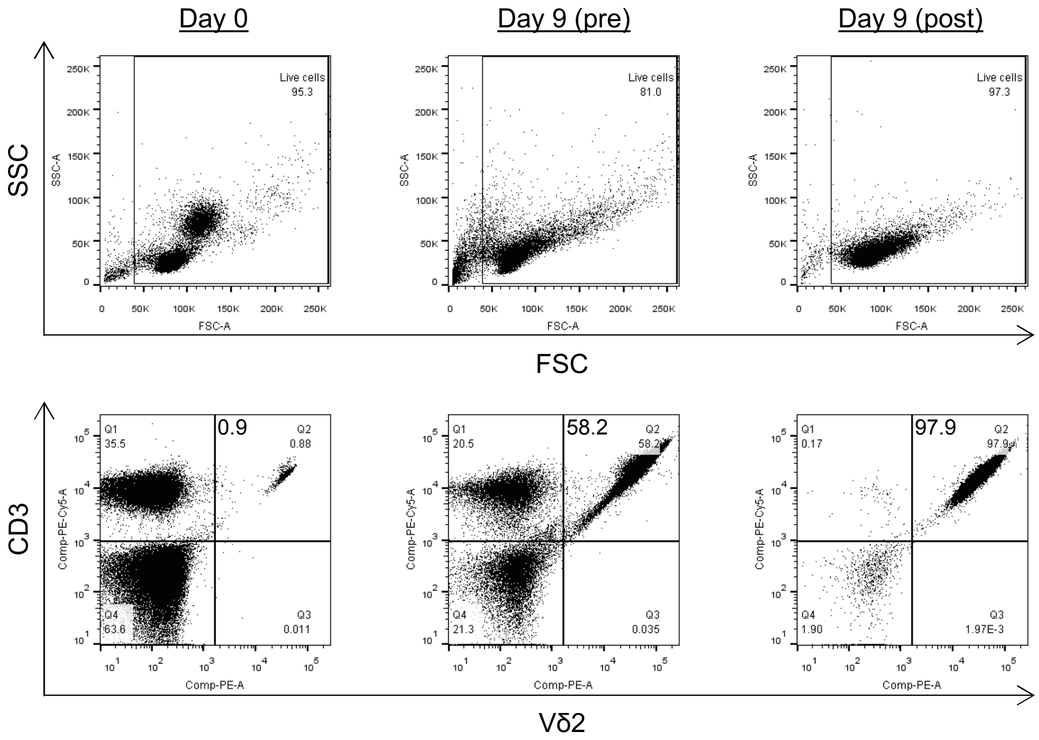

Supplementary Fig. 2: Expansion and isolation of Vδ2⁺ T cells. PBMCs were treated for 9 days with 1μM ZA and 5ng/ml IL-2. Culture medium was replaced with fresh medium containing 5ng/ml IL-2 every 2–3 days. Dead cells and non-γδ T cells were then depleted sequentially using MACS. Flow cytometry was used to measure the percentage of Vδ2⁺CD3⁺ cells at day 0 and 9 pre and post depletion of dead cells and non-γδ T cells. Representative flow cytometry dot plots from one of four donors are shown. Dead cells and debris were excluded based on FSC and SSC, and Vδ2 and CD3 expression was assessed on gated cells. Numbers on the dot plots are percentages of cells contained within the upper right quadrants.

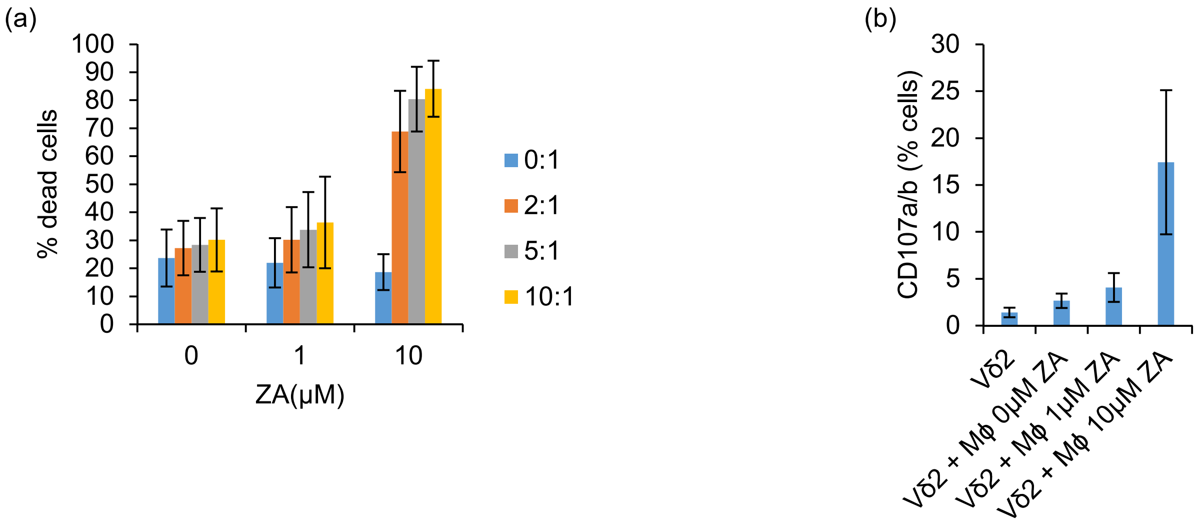

Supplementary Fig. 3: Preliminary optimisation experiments used to determine the concentration of ZA and E:T ratio for cytotoxicity assays. CD14^{+} cells were cultured for 2 hours in serum-free medium and then cultured for 10 days in 10% FBS medium. 25ng/ml IFN-γ was added for the last 48 hours, and 100ng/ml LPS with or without 1 or 10μM ZA was added for the last 18 hours. **(a)** Day 10 Mϕs were washed twice in PBS and then cultured for 20 minutes in PBS containing 1μM CFSE. CFSE^{+} Mϕs were washed three times in complete medium and cultured for 5 hours with or without autologous Vδ2^{+} T cells (generated as described in the materials and methods) at E:T ratios of 2:1, 5:1 and 10:1. Cells were then stained with Zombie-NIR and the percentage of Zombie NIR^{high} cells within CFSE^{+} cells determined by flow cytometry using the gating strategy described in Fig. 2. **(b)** Day 10 Mϕs were washed three times in complete medium and cultured for 5 hours with or without autologous Vδ2^{+} T cells (generated as described in the materials and methods) at an E:T ratio of 2:1. Expression of CD107a and CD107b on Vδ2^{+} T cells was then measured by flow cytometry as described in the materials and methods using the gating strategy described in Fig. 3. For **(a)** and **(b)**, means±SD for three donors are shown and the E:T ratios were based on the number of monocytes seeded at the start of Mϕ differentiation. Results show Vδ2^{+} T cell-mediated killing of Mϕs at the E:T ratio of 2:1 and the 10μM concentration of ZA.

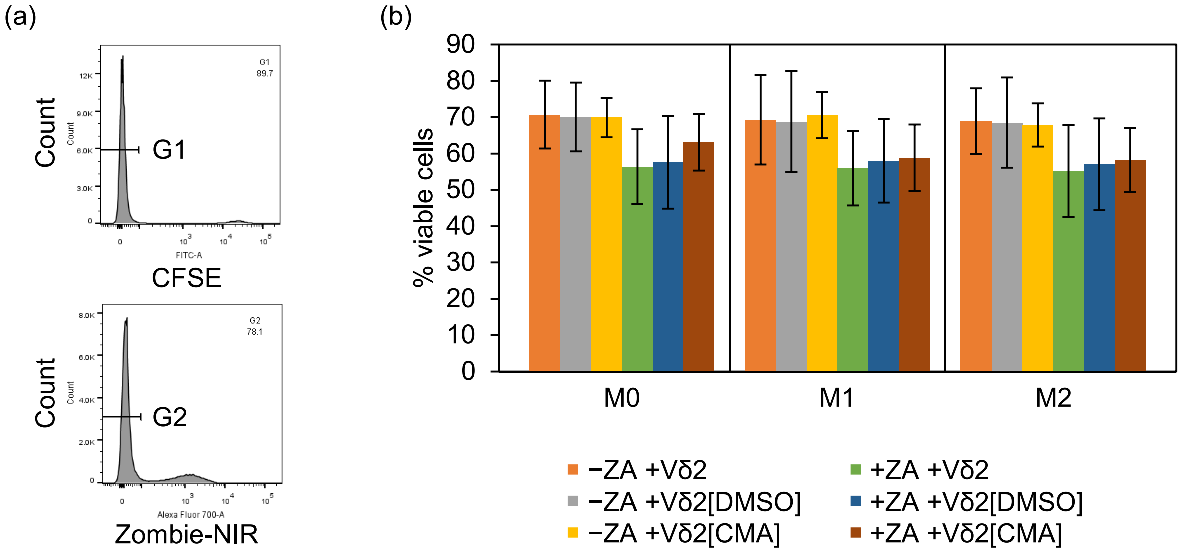

Supplementary Fig. 4: The effect of concanamycin A on the viability of V$\delta 2^+$ T cells. Different gates were applied to the data set shown in Fig. 4. **(a)** Representative flow cytometry plots from one of five donors showing the gating strategy used. $CFSE^-$ cells (i.e. V$\delta 2^+$ T cells) were gated using G1. The percentage of Zombie-NIR^{low} cells (i.e. viable cells) within G1 was then determined using G2. **(b)** Means±SD for five donors.
